# Editorial: The Art and Science of Heroism and Heroic Leadership

**DOI:** 10.3389/fpsyg.2019.00655

**Published:** 2019-03-29

**Authors:** Scott T. Allison, James K. Beggan, Olivia Efthimiou

**Affiliations:** ^1^Department of Psychology, University of Richmond, Richmond, VA, United States; ^2^Department of Sociology, University of Louisville, Louisville, KY, United States; ^3^Department of Digital Media, Murdoch University, Perth, WA, Australia

**Keywords:** heroes, heroism, heroic leadership, transdisciplanary paradigm, heroism science

In July of 2016, the lead editor of this Research Topic, Scott Allison, was privileged to have shared the keynote speaking duties with Peter Le Breton at the First Biennial Heroism Science Conference held in Perth, Australia. The content of these keynote speeches converged on a fundamental truth about studying the rich and elusive phenomenon of heroism: Any adequate understanding of heroic behavior requires a new type of scholarly imagination, one that taps into human artistic sensibilities as much as it does the rigors of scientific inquiry. In an important sense, we were invoking a meta-version of the call to heroic imagination by Franco et al. (2011), who described such imagination “as a mind-set” and “a collection of attitudes” (p. 13) that can steer everyday people toward heroic achievement. In a similar way, Le Breton and I were urging our colleagues in Perth to unleash their own creative mindsets, attitudes, and imaginations in their scholarship on heroism. To acquire a full understanding all of the nuances of heroic action, a deeper trans-paradigmatic approach is needed that integrates the methods and approaches of the arts, the humanities, and the sciences.

The first article in our Research Topic is authored by Kraft-Todd and Rand, who seek to shed light on a centrally important aspect of people's perceptions of heroism. What are the criteria for judging the degree to which a prosocial act is heroic? Kraft-Todd and Rand find that the most heroic acts are not viewed as those benefiting recipients the most; rather, they are acts judged as the most *rare* and *costly* to the hero performing them. The next article in our collection, by Pestana and Codina, also examines people's perceptions of heroes. In doing so, the authors consider three aspects of heroism: the psychological typology of potential leaders, the basic characteristics of people's images of heroism, and the role personal values in shaping hero perceptions. Next, Susan Ross offers an analysis of the hero's personal transformation. Ross shows us how transformation produces an individual who inspires, guides, and protects “something precious.” Her analysis illuminates the forces affecting transformation that may further a broader, collective understanding of this important phenomenon.

In a similar vein, Allison et al. provide an overview of the various types of heroic transformation: mental, moral, emotional, spiritual, physical, and motivational. They also propose a number of activities that can promote heroic metamorphosis as well as several activities that hinder it. Our next article, by Igou et al., offers an analysis of the role of regret in predicting people's motivation to act heroically. Igou et al. find that regret correlates positively with a search for the meaning in life. They also discover that regret predicts heroism motivation and that this effect is mediated through one's search for meaning in life. Our next article, authored by Goldschmied et al., suggests that hailing from humble origins can lead to hero worship. Studying participants from China, Israel, and Japan, these scholars uncover cross-cultural evidence that people's adulation for underdogs is a universal phenomenon. The drive to enforce basic principles of fairness based on competency assessments is shown to be at the root of the choice to support underdog heroes.

Van Tongeren et al. then describe two experiments examining how exposure to superhero images influences both people's tendency to show prosocial behavior and their beliefs about meaning in life. The authors find that people who are primed with superhero images report greater helping intentions relative to a control group, and these helping intentions in turn are significantly associated with increases in people's ability to derive meaning in life. Smyth then argues the philosophical point that heroism involves a “non-selfsacrificial practical necessity.” Smyth approaches the intentional structure of human action from the perspective of embodiment, focusing especially on the predispositionality of pre-reflective skill. The author crafts a philosophical argument that “practical necessity” as an endogenous existential necessity literally embodies socially affirmed values. Smyth's analysis underscores the importance of merging psychological and philosophical perspectives on heroism.

Next, Sanders and van Krieken examine how audiovisual brand stories both invite and enable consumers to enact heroic archetypes. Drawing from Jungian archetypes that dominate the structure of narratives, these scholars distinguish between stories that *show* a hero's journey from stories that not only show but also *tell* one or more hero's journeys. Next, Costello et al. examine the link between psychopathy and heroism. The authors investigate the relationship between psychopathy (focusing on fearless dominance), pride, and prosocial and antisocial behavior. Their results show that fearless dominance is significantly related to pride. Costello et al.'s research illuminates some of the fascinating ways that heroism and villainy intersect.

Our next article, authored by Jones, presents work that nicely showcases the deeper, more intuition-based approach to heroism science that we advocate. Jones invokes mindful meditational practices to further our understanding of the causes and origins of heroism. He notes that practitioners who have attained expertise in mindfulness exercises can develop supernormal capabilities. The mental mastery of one's consciousness allow for the unfolding of the supernormal potential of Buddhist practitioners. Jones reviews the growing empirical literature suggesting that mindfulness may improve physical and emotional health, as well as promote prosocial behavior.

It is our deepest wish that this Research Topic provides you, the reader, with some insights and inspiration about the pinnacle of human behavior. The study of heroism, in all its many expressions, surely requires a creative, non-dualistic, transdisciplinary approach that will be reflected in future theory and research. The editors of the first *Handbook of Heroism and Heroic Leadership* articulated their dream that the growth of heroism science would spawn new research areas and methodologies that were unimaginable in the twentieth century (Allison et al., 2017). We offer the similar hope that the articles contained in this Research Topic reflect a worthy scholarly effort to bring to life the unimaginable.

## Author Contributions

SA, JB, and OE all contributed equally to the ideas contained in this article.

### Conflict of Interest Statement

The authors declare that the research was conducted in the absence of any commercial or financial relationships that could be construed as a potential conflict of interest.

## References

[B1] Allison S. T. Goethals G. R. Kramer R. M. (eds.). (2017). Setting the scene: the rise and coalescence of heroism science in Handbook of Heroism and Heroic Leadership, (New York, NY: Routledge).

[B2] Franco Z. E. Blau K. Zimbardo P. G. (2011). Heroism: a conceptual analysis and differentiation between heroic action and altruism. Rev. Gen. Psychol. 15, 99–113. 10.1037/a0022672

